# Systems biology approaches to toll-like receptor signaling

**DOI:** 10.1002/wsbm.1178

**Published:** 2012-06-19

**Authors:** Alexis Vandenbon, Shunsuke Teraguchi, Shizuo Akira, Kiyoshi Takeda, Daron M Standley

**Affiliations:** 1 Laboratory of Systems Immunology, WPI Immunology Frontier Research Center (IFReC), Osaka UniversitySuita, Osaka, Japan; 2 Laboratory of Host Defence, WPI Immunology Frontier Research Center (IFReC), Osaka UniversitySuita, Osaka, Japan; 3 Department of Microbiology and Immunology, Graduate School of Medicine, Osaka UniversitySuita, Osaka, Japan; 4 Laboratory of Mucosal Immunology, WPI Immunology Frontier Research Center (IFReC) Osaka UniversitySuita, Osaka, Japan; 5 Research Institute for Microbial Diseases, Osaka UniversitySuita, Osaka, Japan

## Abstract

Toll-like receptor (TLR) signaling pathways constitute an evolutionarily conserved host defense system that protects against a broad range of infectious agents. Modeling of TLR signaling has been carried out at several levels. Structural models of TLRs and their adaptors, which utilize a small number of structural domains to recognize a diverse range of pathogens, provide a starting point for understanding how pathogens are recognized and signaling events initiated. Various experimental and computational techniques have been used to construct models of downstream signal transduction networks from the measurements of gene expression and chromatin structure under resting and perturbed conditions along with predicted regulatory sequence motifs. Although a complete and accurate mathematical model of all TLR signaling pathways has yet to be derived, many important modules have been identified and investigated, enhancing our understanding of innate immune responses. Extensions of these models based on emerging experimental techniques are discussed. © 2012 Wiley Periodicals, Inc.

## INTRODUCTION

**FIGURE 1:**
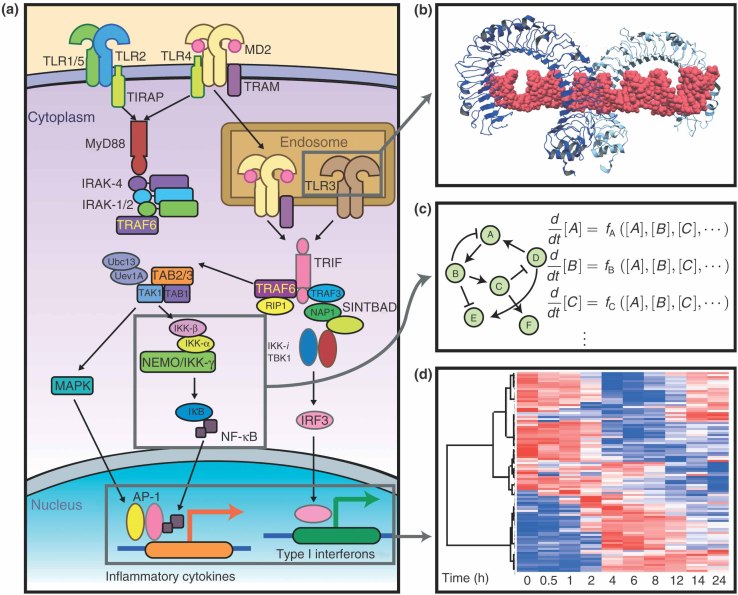
Multi-scale modeling of toll-like receptor (TLR) pathways. (a) The MyD88- and TRIF-dependent pathways are illustrated. Reprinted with permission from Ref 2 Copyright 2010 Elsevier. (b) The X-ray structure of TLR3 leucine rich repeat (LRR) domains bound to double-stranded RNA. (c) A mathematical model of a hypothetical signaling network between components A–F. (d) A heatmap of gene expression values at 10 time points.

Pattern recognition receptors (PRRs) that specifically bind to evolutionarily conserved pathogen-associated molecular patterns (PAMPs) play a key role in host defense. Of the PRRs, the toll-like receptors (TLRs) are the best characterized, both in terms of the PAMPs they recognize and the corresponding pathways that are activated in response to their binding. To date, 10 TLRs have been identified in humans, each of which has a homolog in mouse. Identifying the ligand specificity and downstream pathways of each TLR (or combination, as TLRs function as dimers) has been the subject of intense research for the last 15 years.^1^,^2^ Two main TLR signaling pathways have been identified: the MyD88 (myeloid differentiation factor 88)-dependent^3^ and the TRIF (TIR-domain-containing adaptor protein-inducing IFN-*β*)-dependent^4^ pathways. The MyD88-dependent pathway is activated by all known TLRs except TLR3, and leads to the production of proinflammatory cytokines. TLR3 and TLR4 can activate the TRIF-dependent pathway, which leads to the production of type I interferons (IFNs) against viral infection. Hundreds of proteins have been characterized as players in the innate immune response, and thousands of genes whose expression levels change upon TLR stimulation have been identified. Many protein–protein interactions have been determined for TLR signaling molecules as well.^5^ However, a successful immune response requires the coordinated interaction between all of its parts, from a molecular to an intercellular level. Reconstructing and reproducing this system-level behavior are a daunting task, and one that is impossible to accomplish without the development of multilevel and multi-timescale computational frameworks. In this review we will summarize the current models, focusing on structure, signal transduction, and gene expression analysis (Figure 1).

## STRUCTURAL MODELING OF TLRs AND THEIR ADAPTORS

**FIGURE 2:**
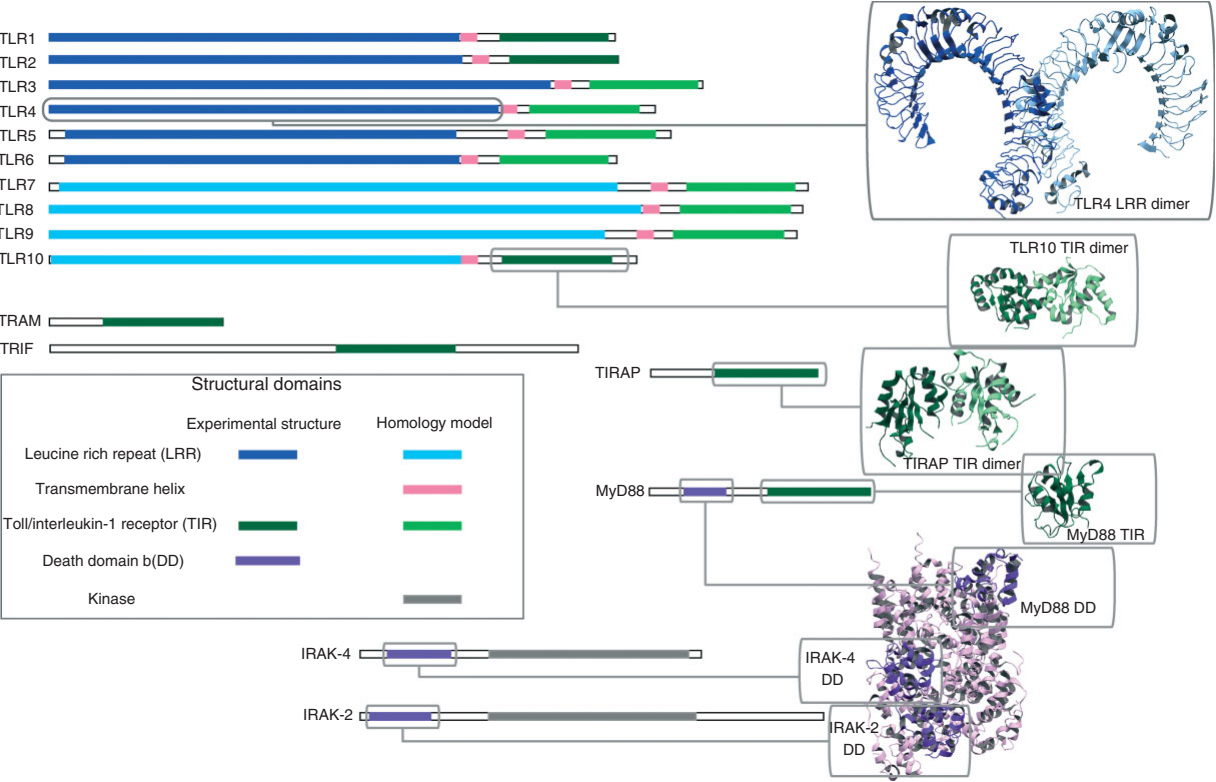
Structural domains of toll-like receptors (TLRs) and their adaptors. The canonical domains of TLRs and their adaptors are shown as 1D bar graphs with the domains drawn to scale. Darker colors indicate experimentally determined structures while lighter colors indicate domains that can be modeled by homology. The cartoon representations of several representative structures are drawn to scale: TLR4 leucine rich repeat (LRR) dimer,^15^ TLR10 TIR (interleukin-1) receptordimer,^16^ TIRAP TIR dimer,^17^ MyD88 TIR,^18^ and myddosome complex.^9^ In the cartoon representations, dark/light shades are used to distinguish individual chains in dimers.

Homology modeling (Box 1) has played an important role in the analysis of TLR signaling pathways because a number of structural domains reoccur in various contexts. As shown in Figure 2, each of the TLRs contains an N-terminal leucine rich repeat (LRR) domain, followed by a transmembrane helix and a cytoplasmic C-terminal toll/interleukin-1 receptor (TIR) domain. The cytoplasmic TIR domains, in turn, bind TIR-containing adapter molecules. In the case of the MyD88-dependent TLR4 signaling pathway, for example, a TLR4 TIR domain homodimer interacts directly with the TIR-containing adaptor TIRAP (TIR-associated protein; also known as Mal).^6^,^7^ TIRAP, in turn, interacts directly with MyD88,^8^ which contains both a TIR domain and a death domain (DD). The MyD88 DD interacts with DD-containing IL-1R-associated kinase-4 (IRAK-4), which interacts with DD-containing IRAK-2. Current evidence supports a model in which each of these pairwise interactions occurs within a large signaling complex.^9^

**BOX 1**

**COMPUTATIONAL METHODS IN SYSTEMS BIOLOGY**

*Homology modeling:* Known protein structures are used as templates for predicting the structure of a query protein sequence. Such ‘template-based’ methods are regularly accessed in the Critical Assessment of Techniques for Protein Structure Prediction.^10^

*Protein docking:* The quaternary structures of protein complexes are predicted from the tertiary structures of the individual component proteins. Challenges include integration of disparate experimental restraints, and predicting conformational changes that occur upon complex formation.^11^

*Ordinary differential equations (ODEs)*: The dynamics of cellular systems are modeled by using biochemical reaction equations such as mass action or Michaelis-Menten kinetics with respect to molecular concentrations. Here, stochasticity inside a cell or heterogeneity of cell population is not considered and systems evolve deterministically.

*Stochastic modeling:* In a cell, there are several sources of ‘noise’, which may cause heterogeneity at the population level. Such noise can be modeled by introducing random variables or Monte-Carlo simulation.^12^

*Flux balance analysis (FBA):* Once stoichiometric coefficients of a biochemical network are known, they impose a constraint on the possible configuration of fluxes at its steady state. FBA determines the optimal fluxes by maximizing a suitably chosen objective function, without requiring knowledge of each kinetic parameter.^13^

*Position weight matrix (PWM):* PWMs are widely used probabilistic models for sequence motifs in computational biology.^14^ In the context of this review, PWMs are used as a model of the DNA-binding preferences of transcription factors, and can be used to predict transcription factor binding sites (TFBSs).

### LRR Domain Models

The crystal structures of the LRRs from the TLR1/ TLR2^19^ and TLR6/TLR2^20^ complexes bound to lipopeptide, the TLR4 dimer bound to myeloid differentiation protein-2 (MD-2) and lipopolysaccharide (LPS),^15^ the TLR3 dimer bound to dsDNA,^21^ and the TLR5 dimer bound to flagellin^22^ have been solved. As a result, it has been possible to predict the structures of the remaining TLRs as well as their ligand binding residues. For example, homology modeling of the TLR9 LRR domain revealed that putative nucleotide binding sites in addition to a number of conserved cystine residues that were shown by site-directed mutagenesis to be essential for TLR signaling.^23^ Several studies have investigated the ligand binding properties of other TLRs computationally.^24^–^26^ In one such study, homology modeling in combination with generation of LRR/TIR chimeras showed that, like TLR1, TLR10 can bind to TLR2 and has a putative lipopeptide binding site, but that the downstream signaling pathways facilitated by interaction with the TIR domains differ.^24^

### TIR Domain Models

A complete structural level understanding of the TIR–TIR interactions that mediate the specific downstream signaling pathways remains elusive, although a number of important advances toward this goal have been made recently. For example, homology modeling indicated that the homodimerization interface of the TLR4 TIR domains is similar to that of TLR10 (Figure 2), which involves pairing of two so-called BB loops, and that this homodimer interface creates a new interface for TIRAP or TRAM (TRIF-related adaptor molecule) binding.^6^,^7^ However, one protein docking study (Box 1) resulted in TIRAP binding to two symmetry-related sites on TLR4,^7^ whereas a more recent model supported by the sequence conservation and reporter assays in mammalian cells, places two TIRAP molecules adjacent to each other.^6^ The crystal structure of TIRAP has been solved, and is predicted to form a twofold symmetric homodimer.^8^ However, two models of TIRAP-MyD88 heterodimerization have been proposed: one in which two TIRAP TIR molecules interact with a single MyD88 TIR domain,^18^ and the other in which two MyD88 TIR domains bind to opposite sides of the TIRAP homodimer.^8^ The latter model is consistent with the observation that residue D96 in TIRAP and R196 in MyD88 are important for the MyD88-TIRAP interaction.^8^ The difficulty in experimentally determining quaternary structures of TIR domain complexes is partially due to their weak binding affinities. This, in turn, means that computational approaches, such as protein docking, in combination with site-directed mutagenesis and protein–protein interaction assays, are expected to play an important role in elucidating the structures of transient signaling complexes.

### Death Domain Models

Recently, an X-ray crystallography study revealed the structure of a helical myddosome complex composed of the DDs of MyD88, IRAK-4, and IRAK-2.^27^ The myddosome contains 4-6 MyD88 DDs, and 4 DDs each from IRAK-4 and IRAK-2. While it has not yet been determined whether the helical myddosome is present *in vivo*, the authors argue that the 4-6 MyD88 DDs in the myddosome structure suggest a higher-order clustering of TLR dimers, possibly localized on lipid rafts.^9^ It is expected that further experiment along with structural modeling will clarify what implications the proposed myddosome complex has for spatial arrangement of upstream TIR and LRR complexes.

As the above examples show, structural models provide a framework for understanding the details of macromolecular interactions in terms of their geometry and physical properties. In order to understand the biology of such interactions we need look at larger systems of molecules.

## INFERENCE OF TLR SIGNAL TRANSDUCTION NETWORKS

### Mathematical Models of Signal Transduction Networks

**FIGURE 3:**
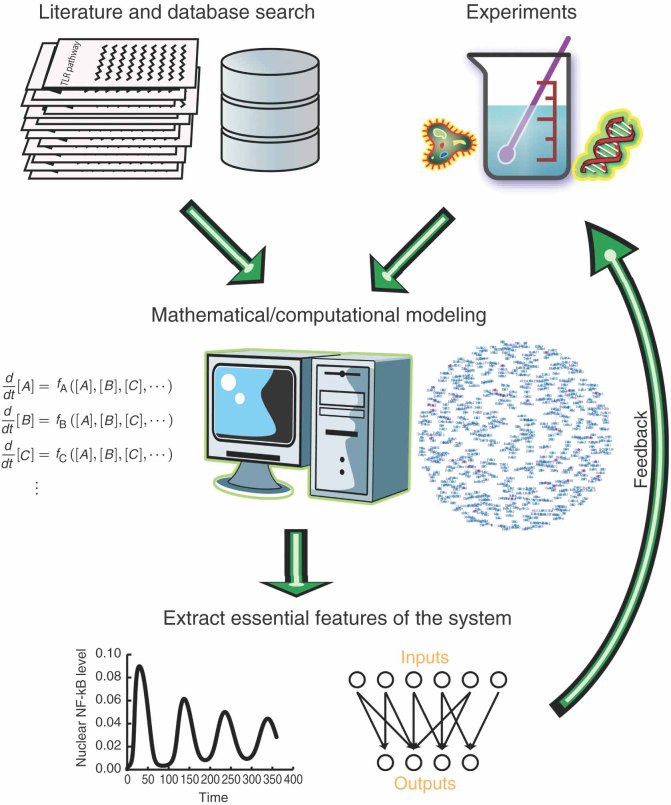
Schematic picture of mathematical modeling approaches to toll-like receptor (TLR) signal transduction networks. In order to construct mathematical models, several kinds of data, such as network topology and biochemical parameters, are required. Those data must be collected or inferred from literature, databases, or experiments. Mathematical models allow us to predict system behavior under different conditions based on assumed rules (or laws). Essential features of the system, such as oscillatory behavior caused by the strong negative feedback loops of IkBa or input–output relationships among TLR receptors and transcriptional output, can be extracted through such models. Predictions can be validated using further experiments, thus enhancing our knowledge of the system.

Mathematical modeling of TLR signal transduction networks allows us to ignore the internal details of each macromolecule in order to focus on their system-level interactions (Figure 3). Early work in this direction focused on integrating data from small-scale experiments. For example, Hoffmann and coworkers^28^ constructed a biochemical model of the NF-kB (nuclear factor *κ*-light-chain-enhancer of activated B cells)/IkB (NF-kB inhibitor) module, which is an important component of the downstream of TLR pathway. Their model is a large set of ordinary differential equations (ODEs, Box 1) based on biochemical parameters derived from cell population averages. They showed that, among three isoforms of IkB (IkB*α*, IkB*β*, and IkB*ε*), IkB*α* participates in a strong negative feedback loop, which results in oscillatory behavior of NF-kB upon tumor necrosis factor-*α* (TNF*α*) stimulation, while the other two isoforms dampen the oscillation. They have also observed similar oscillatory behavior of NF-kB upon LPS stimulation in MyD88 or TRIF deficient mice.^29^ In subsequent papers,^30^,^31^ they hypothesized that the temporal patterns of IKK (IkB kinase) activation, which leads to the phosphorylation of IkB and the activation of NF-kB, encode ligand-specific information of upstream signaling. On the basis of this hypothesis, they have modeled the NF-kB activation upon TNF*α* or LPS stimulation based on experimentally measured IKK activity patterns. These examples illustrate that it is possible to infer the function of molecules through mathematical models, which would be difficult if not impossible experimentally. Details of such models and related works were nicely summarized in Ref 32. It is worth noting, although, that predictions by ODE models in general largely depend on the chosen kinetic parameters. In the above models, the authors made great efforts to collect data from the literature as well as through their own experiments. However, it is debatable whether, in the context of ODEs, one can simply integrate data obtained in different cellular contexts or parameters obtained from different models. Nevertheless, even imperfect ODE models provide the basis for further refinement. It is equally important to seek modeling methods that tolerate integration of data and parameters from various contexts, as discussed in Ref 33. Alternatively, approaches that do not require predetermined kinetic parameters can yield important insights, as discussed below.

Another early milestone in systems-level analysis of TLR signaling networks was carried out by Oda and Kitano in 2006.^34^ They constructed a comprehensive map of known TLR signaling components based on literature searches. The map revealed a bow-tie structure, where divergent input signals flow into the MyD88 ‘core’ of the network and branch out to multiple components, with much crosstalk with a few collateral pathways. More recently, Li and coworkers^35^ analyzed a large-scale TLR signaling pathway using flux balance analysis (FBA, see Box 1), which has origins in the field of metabolic networks. They modified the original Oda-Kitano TLR map to meet FBA conditions and identified simple input–output relationships based on ligand/receptor and transcription/ anti-pathogen activities, respectively, by optimizing the signaling flux with respect to the output flux. This procedure significantly reduced the complexity of the full network consisting of 752 distinct chemical species and 909 reactions into 41 relationships between 14 receptors and 6 outputs. They further simplified the network by identifying redundant pathways and identified eight critical biochemical reactions, which specifically affect the pathways of reactive oxygen species (ROS), IL-1-induced NF-kB activation and MyD88-mediated NF-kB/AP-1 activation. Because it is not yet clear how analyses based on steady-state behavior like FBA apply to the dynamics of TLR signal transduction networks, it will be important to validate such predictions experimentally. In addition, some of the discussion in Ref 35 was apparently based on an earlier misidentification of TLR2 as an LPS receptor, which has been subsequently shown to be a result of contamination. Thus, in spite of the great effort made in curating the original network, this example indicates that continuous collaboration between experts in computational and immunology fields is very important.

### Combining Gene Expression and Regulatory Sequence Motifs

While the above network models are based on known components and topologies of TLR signal transduction, it is of great interest to infer the yet unknown components and regulatory relationships by computational approaches. A strategy often used is to predict shared regulatory motifs in the regulatory regions of co-expressed genes (Box 2). Despite the apparent simplicity of this approach, it is hampered in practice by the low specificity and sensitivity of transcription factor binding site (TFBS) prediction using position weight matrices (PWMs)^36^ (see Box 1). Nevertheless, because experimental identification of regulatory sites is expensive and labor intensive, computational predictions are often used to provide a first hint or hypothesis, which can subsequently be tested by wet lab experiments. An example of one such study started from sets of genes with similar expression profiles in macrophages after TLR stimulation.^37^ The authors next scanned promoter sequences of these genes with a set of PWMs, and identified possible regulatory relationships between TFs and clusters of co-regulated genes. These relationships were subsequently combined with additional gene expression data in order to predict causal relationships between regulators and target genes. An important detail was their use of time-lagged correlation between the expression of TFs and their candidate target genes, allowing for the prediction of causal TF-target relationships. Among the important genes they identified, known regulators, such as NF-kB, interferon regulatory factors (IRFs), and AP-1, were found as well as a previously unidentified regulator, TGIF1.

**Box 2**

**BASIC REGULATORY NETWORK INFERENCE STRATEGIES**

**FIGURE 4:**
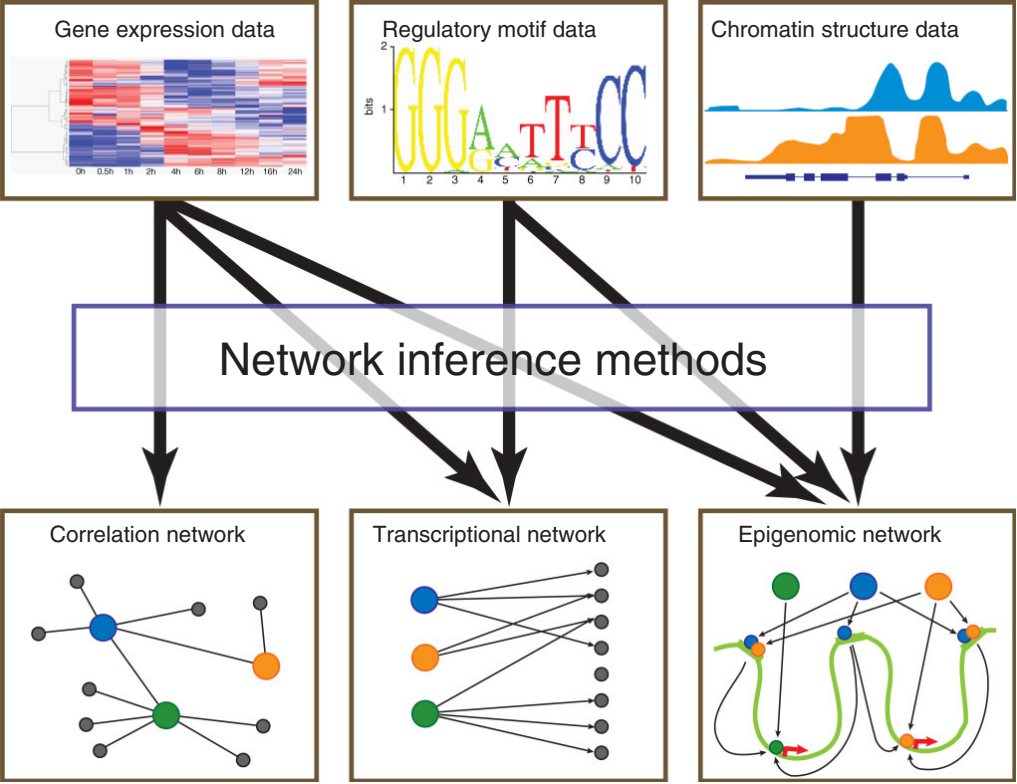
Levels of complexity in network inference. By integration of additional types of data with expression data, the inference of increasingly complex networks becomes possible. Regulatory motif data can be used to add directionality to gene expression-based networks, while increasing amounts of epigenetic data will in the near future allow us to construct genome-wide networks including distal regulatory enhancers as well as traditional network components.

Network inference approaches utilize large-scale sequence and expression data to reconstruct biological networks. A relatively simple approach to network inference is based on the assumption that co-expressed pairs of genes have some level of interaction (Figure 4). Typically, a pairwise comparison of the expression profiles of all genes is made, and a network is constructed where each pair of significantly correlated genes is connected by an edge. Importantly, correlation of expression does not necessarily imply a causal relationship, and is not able to distinguish direct interactions from indirect ones. A number of strategies have been developed that attempt to solve the above problem, such as the use of time-lagged correlation of expression, and various integrative approaches.^38^ The latter typically use transcription factor binding sites in the regulatory regions of target genes to predict direct regulatory interactions (Figure 4). Although a number of successful applications to mammalian data have been reported,^37^,^39^ noisy expression data and the relatively small size of regulatory sites make network inference a non-trivial problem. In addition, the use of mRNA levels as estimator for gene activity introduces an additional layer of complexity, as regulation of translation, post-translational modifications, and cellular localization are known to play an important role in the regulation of gene activity.^40^,^41^ Here, further advances in measuring protein abundance and post-translational modifications,^42^ such as phosphorylation^43^ will allow for a better understanding of TLR signaling.

Rather than attempting to explain the regulation of transcription on a large scale, some studies have performed detailed dissection of a small set of genes or regulatory sites. In one such study, Leung and coworkers focused on genes that are under the regulation of two NF-kB binding sites.^44^ They found that in these genes both sites are required for the activity of the gene, and that swapping the sites alters the NF-kB family members that bind to them. More importantly, they found that the combination of the two sites affects which coactivator binds to the bound NF-kB dimer, and that even a single nucleotide within the NF-kB site can change the cofactor specificity. In another study, Giorgetti and colleagues showed that clusters of NF-kB binding sites could be used in a noncooperative way to process increasing levels of NF-kB into gradual increments of transcriptional response.^45^ This result was in sharp contrast with the widely accepted view that graded increases in TF concentration result—through cooperative binding—in a digital transcription initiation signal. These two findings are indicative of a wide variety of features that contribute to different transcriptional responses.

### Network Inference from Large-Scale Perturbations

A number of studies have analyzed the activity of transcriptional regulators by systematic perturbation experiments. Recently, for example, Amit and coworkers reconstructed the regulatory relationships among transcripts whose expression levels depend on TLR stimulation.^46^ They observed transcriptional expression levels for 118 predetermined target genes 6 h after LPS stimulation in dendritic cells. The cells were independently perturbed using small hairpin RNA (shRNA) for 144 candidate regulators. The resulting gene expression levels were used to define statistically significant activating and repressing relationships between regulators and target genes. As a result, they identified 1728 activations and 594 repressions. Although their results likely contain a significant number of indirect regulations, their study nevertheless quantified interactions between components in the inflammatory and antiviral programs of dendritic cells with unprecedented breadth. This is an example where, in the case of DNA-binding proteins, sequence analysis or ChIP-seq experiments, discussed below, might help to distinguish between direct and indirect interactions. Using a similar perturbation approach, along with predicted TF activities, Suzuki and coworkers examined 52 TFs in human myeloid leukaemia cells.^47^ In these perturbation studies, genome-wide data were first used to select a smaller set of representative genes. These representatives included candidate regulators for perturbation experiments, and also a subset of genes for which expression changes will be measured. Narrowing down the number of genes of interest is an important step because it maximizes the information obtained from a given experiment. Although cell types and stimuli were different, the results of both studies suggest that the gene expression in a single immune cell type is controlled by a substantial number of core regulators and additional fine-tuners.

### Future Perspectives

Although most studies on the regulation of transcription have focused on the roles of TFs and their binding sites, additional levels of regulation exist, one being the structural state of the chromatin (Figure 4). Transcription can be regulated on an epigenetic level by various mechanisms (see reviews 11, 48), and it is likely that some TFs are associated with different epigenetic changes. Importantly, it has become clear that primary (or immediate-early) response genes and secondary response genes in TLR signaling differ fundamentally in their chromatin structure, as well as their tendencies to have preassembled RNA-polymerase II at their promoters, dependence on chromatin remodeling for induction, and association with CpG islands.^49^–^51^ Although a number of studies have elucidated interactions between TFs and histone modifiers,^52^–^56^ in general, the causal relationships between these features are still unclear. Nevertheless, our current understanding suggests that any approach aiming at modeling or explaining the dynamics of gene expression during the immune response should try to incorporate the fact that several classes and subclasses of regulatory regions exist, and that they are likely to be under the control of fundamentally different regulatory mechanisms (see review 48).

Recent studies have attempted to combine chromatin structure and histone modification data with the analysis of regulatory networks. In general, these approaches aim to use epigenetic features as a measure of accessibility of DNA sequences or activity of genes, and to use this as prior knowledge in the discovery of regulatory motifs. One example is the study by Ramsey et al.^57^ who focused specifically on macrophages. After combining ChIP-seq data for a number of TFs with histone acetylation (HAc) data, the authors observed that TFBSs often occur within local minima of HAc ChIP-seq signals within HAc-rich regions. Based on this observation they defined a ‘valley score’ and they showed that the use of this score in combination with PWM scores could improve TFBS prediction accuracy. The improvement was variable from TF to TF though, suggesting that depending on the biological function of the TF, different epigenetic features might lead to better predictions. Approaches such as CENTIPEDE^58^ and simpler methods^59^ that aim to computationally predict TF binding events using a limited amount of experimental data yet with an accuracy similar to that of ‘gold-standard’ ChIP-seq experiments are therefore likely to continue to play an important role in system-level analyses of transcriptional regulation.

## CONCLUSION

Computational modeling has played an important role in the study of TLR signaling. Since many of its components are shared between organisms as diverse as mice, insects, and worms, sequence homology has guided many pioneering experiments that have revealed key biochemical functions in these pathways. Computational analysis of the macromolecular structures along with site-directed mutagenesis has provided insight into the mechanism of signaling pathways in normal and diseased states. Accurate mathematical modeling of signal transduction dynamics is a challenging goal due to our incomplete knowledge of the components and their interactions. However, the general topology of the TLR signaling network in mammals has been established. Gene expression data in parallel with controlled perturbations will enable current models to be continuously refined. Extensions of these models wherein structural information is integrated with network-based signaling models are expected to provide a more quantitative description of TLR signaling in the future. The increasing number of public databases, such as the innateDB,^60^ ImmGen^61^ and Macrophages.com,^62^ and tools^63^,^64^ will enable further refinement by facilitating data sharing and interpretation, and establishing standards. Finally, it must be acknowledged that immunology is still very much an experimental discipline. The emergence of new experimental techniques, especially those that quantify gene and protein expression levels, as well as epigenetic and post-translational modifications, is expected to add depth to our understanding. However, we are convinced that in order to understand the immune response on a system's level, and the interactions between its various parts, the future contribution of computational methodologies will be invaluable. We believe that the studies we have discussed above will be a foundation for future developments.
